# Design and Experiment of Combined Infrared and Hot-Air Dryer Based on Temperature and Humidity Control with Sea Buckthorn (*Hippophae rhamnoides* L.)

**DOI:** 10.3390/foods12122299

**Published:** 2023-06-07

**Authors:** Zhihua Geng, Mengqing Li, Lichun Zhu, Xiaoqiang Zhang, Hongbo Zhu, Xuhai Yang, Xianlong Yu, Qian Zhang, Bin Hu

**Affiliations:** 1 College of Mechanical and Electrical Engineering, Shihezi University, Shihezi 832003, China; gzhjdxy@163.com (Z.G.); limengqing0305@163.com (M.L.); zhulichun0204@126.com (L.Z.); 18534935245@163.com (X.Z.); 15042020742@163.com (H.Z.); zq_mac@shzu.edu.cn (Q.Z.); 2 Xinjiang Production and Construction Corps Key Laboratory of Modern Agricultural Machinery, Shihezi 832003, China; 3 Shandong Academy of Agricultural Sciences, Jinan 250100, China; 4 Engineering Research Center for Production Mechanization of Oasis Special Economic Crop, Ministry of Education, Shihezi 832003, China

**Keywords:** combined infrared and hot-air dryer, temperature and humidity control, numerical simulation, structure optimization, sea buckthorn

## Abstract

A drying device based on infrared radiation heating technology combined with temperature and humidity process control technology was created to increase the drying effectiveness and quality of sea buckthorn. Based on the conventional k-turbulence model, the velocity field in the air distribution chamber was simulated using COMSOL 6.0 software. The airflow of the drying medium in the air distribution chamber was investigated, and the accuracy of the model was verified. Given that the inlet of each drying layer in the original model had a different velocity, the velocity flow field was improved by including a semi-cylindrical spoiler. The results showed that installation of the spoiler improved the homogeneity of the flow field for various air intakes, as the highest velocity deviation ratio dropped from 26.68% to 0.88%. We found that sea buckthorn dried more rapidly after being humidified, reducing the drying time by 7.18% and increasing the effective diffusion coefficient from 1.12 × 10^−8^ to 1.23 × 10^−8^ m^2^/s. The L*, rehydration ratio, and vitamin C retention rate were greater after drying with humidification. By presenting this hot-air drying model as a potential high-efficiency and high-quality preservation technology for sea buckthorn, we hope to advance the development of research in the sea buckthorn drying sector.

## 1. Introduction

Agricultural products made from sea buckthorn (*Hippophae rhamnoides* L.) have important roles in both food and medicine. The plant contains flavonoids and polyphenols and is nutrient dense. It aids in the treatment of heart disease, hypertension, and hyperlipidemia, improves blood circulation, disperses blood stasis, clears heat, and halts diarrhea [1,2]. Fresh sea buckthorn contains up to 80% moisture and has abundant vitamins and unstable active polyphenols that are easily oxidized, making it quite perishable [3,4]. It should not be stored for an extended period after harvesting because it ferments and quickly degrades at room temperature, causing loss of economic and therapeutic potential. Therefore, preservation methods are necessary to maintain the usefulness of sea buckthorn, and one common method of preservation is drying [5].

Air convection and heat conduction are used in hot-air drying as the drying medium to conduct heat exchange. Sea buckthorn fruit dries fastest when submitted to hot-air drying [6,7]. Although hot-air drying equipment has a straightforward design and is inexpensive, heat must be transferred from the surface of the material to its internal matter through heat conduction. As a result, drying times are lengthy, nutrient components are severely oxidized, and quality suffers [8]. Infrared drying works by converting a portion of the energy into the erratic thermal motion energy of molecules, which warms the material and removes moisture to complete the drying process [9,10]. Combining infrared and hot-air drying increases the drying effectiveness by taking advantage of the capabilities of each technology. However, rapid moisture evaporation from a material’s surface during drying may cause the material’s surface to harden and prevent water from evaporating during the drying stage. Recent developments in drying technology have led to improvements in temperature and humidity control [11]. A high relative humidity during the initial drying stage causes the internal temperature of a material to rapidly increase. Once the relative humidity in the environment has been reduced, the drying rate increases, and the problem of the surface developing a crust from rapid drying can be avoided [12].

Combined infrared and hot-air drying technology has been used extensively in the drying and processing of various fruits and vegetables to significantly reduce drying time. However, sea buckthorn is a berry with a dense waxy layer on the skin, similar to grapes, wolfberries, and blueberries, which hinders moisture diffusion from the inside to the outside of the fruit. Therefore, new technologies must be combined to improve moisture diffusion efficiency. The drying process uses hot air, and at a given temperature, the enthalpy of the drying medium rises with increasing humidity [13]. However, at low relative humidity, a material warms up more slowly; conversely, at the high enthalpies of wet air, a material warms up quickly over a shorter period [14]. Compared to the conventional single low-humidity hot-air drying technology, reducing the drying temperature and maintaining a high relative humidity can reduce the heating time, as the higher relative humidity at the beginning of drying can cause the material’s internal temperature to rise quickly [15]. This improves the drying rate and prevents crust formation on the material’s surface due to fast drying. However, temperature and humidity control-related machinery or manufacturing processes for the sea buckthorn berry have yet to be reported.

The homogeneity of the drying apparatus has recently received the attention of researchers. To address the issue of uneven airflow in the drying room of a conventional solar dryer, Senay et al. [16] used the computational fluid dynamics (CFD) method to investigate the transient flow of dry air and evaluate the performance of the air dispenser in the dryer. To enhance the functionality and caliber of solar dryer products, an airflow distribution system was created based on simulations to determine the uniform distribution of various airflow types. To address the uneven drying of materials due to the distribution of airflow in a conveying dryer, Sabyasachi et al. [17] conducted a CFD simulation to examine and validate the distribution of airflow in the drying chamber; drying uniformity was enhanced by the addition of a spoiler. To analyze the impact of the drying wind speed and temperature distribution in the drying chamber, Daza-Gomez et al. [18] simulated the uniformity of the drying chamber of a heat-pump dryer by considering the drying wind speed and temperature distribution. The drying chamber was altered to ensure uniformity based on the simulation results. Shi et al. [19] investigated the non-uniformity issue in the heat-pump drying procedure. They discovered that the airflow uniformity at the fan outlet and the partition opening was relatively poor through simulations with the porous medium model and multi-reference frame model. Therefore, these authors designed an air baffle structure and used numerical calculations to determine the ideal separation size of the air baffle. The results demonstrated the capability of the model to accurately estimate air temperature and relative humidity.

In this study, we developed combined infrared and hot-air drying equipment based on temperature and humidity control suitable for drying sea buckthorn, with the goal of improving the drying efficiency and effectiveness for sea buckthorn. The impacts of key parameters on sea buckthorn drying and material quality were also examined. We studied the relationship between drying uniformity and the spatial distribution of air velocity, medium temperature, and medium humidity; we used COMSOL for numerical simulation; we studied the distribution law of the flow field; we explored the uniform distribution law between and within drying layers during the drying process; and we developed an effective quality preservation strategy to promote the development of the sea-buckthorn drying industry.

## 2. General Plan Design

### 2.1. Analysis of Design Requirements

During the sea buckthorn drying process, the drying device must accurately control the temperature (°C), humidity (%), and wind speed (m/s) of the medium in the drying room. Therefore, the mechanical structure and control system are two components of the combined infrared and hot-air drying device based on temperature and humidity control. The mechanical components of the drying equipment include a drying chamber, humidifier, heater, and hot-air circulation system. It is necessary to implement real-time monitoring of the temperature and humidity in the drying machine and the internal sea buckthorn temperature. This further increases the monitoring range and accuracy of the sea buckthorn temperature because the changes in air temperature and humidity in the drying room are closely related to the drying process.

### 2.2. Working Principle

When the dryer is in operation, the human–computer interface controls the temperature and humidity of the drying chamber. It also activates a carbon fiber infrared radiation electric heating plate. Air enters the air duct from the right rear air inlet, is heated by an electric heating tube, and exits the air duct through the air inlet on the left side of the material tray entering the drying chamber. The carbon fiber infrared radiation plate of the material tray heats the sea buckthorn by releasing the infrared radiation. The airflow returns to the air distribution chamber through the air exit on the right side after the sea buckthorn and airflow have successfully exchanged heat. The fan achieves waste heat recovery and lowers the energy consumption of the drying device by rotating the hot air in the air duct into the fan for secondary heating.

### 2.3. Dryer Design

**Figure 1:**
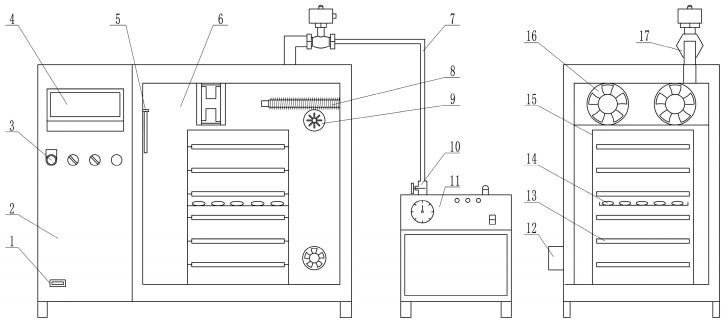
Schematic diagram of combined infrared and hot air-drying device based on temperature and humidity control. 1. USB interface. 2. Control cabinet. 3. Indicator. 4. Human-machine interface 5. Temperature and humidity sensor. 6. Drying chamber. 7. Humidification pipe. 8. Electric heating pipe. 9. Air inlet. 10. Steam valve. 11. Steam generator. 12. Exhaust fan. 13. Infrared heating version. 14. Sea buckthorn. 15. Material rack. 16. Axial fan. 17. Solenoid valve.

Figure 1 shows the general layout of the combined infrared and hot-air drying equipment based on temperature and humidity control. The drying apparatus had a total of 304 stainless steel walls on the inside and outside. Ultrafine glass fiber insulation cotton, which has low thermal conductivity and potential flame resistance, was used to make the insulation sandwich in the center of the box. To guarantee the durability and dependability of the box structure, square tubes were installed within the drying box as a frame. Five drying levels, spaced equally from top to bottom, comprised the inside of the drying chamber.

#### 2.3.1. Infrared Heating Plate Selection

The heat resistance, fire resistance, and insulation of the carbon fiber infrared radiant heating plates were found to be suitable [20]. The upper material was made of polyethylene terephthalate resin, the lower base was made of epoxy resin glass fiber cloth, and the heating layer was a layer of carbon fiber conductive paper. The carbon fiber infrared radiation heating panel stimulated far infrared rays under 220 V excitation, and the energy conversion efficiency was high. A carbon fiber infrared radiant heating plate with a power of 0.30 kW and a size of 280 mm × 240 mm was chosen because it provided heat and allowed for easy dismantling.

#### 2.3.2. Heating Tube Selection

Ovens and other equipment frequently have electric heating tubes owing to their excellent efficiency, small size, light weight, durability, quick heating, and low cost [21]. To effectively avoid the issue of the fan being too close to the heat source, which may damage the circulating fan, the electric heating tube was positioned in front of the air inlet on the top right by screws. Between the heating and drying chambers, there was an air supply line that could prevent the sea buckthorn from being damaged by the high temperatures produced by the heating pipe. A single finned heating tube with a metal radiator on its surface, which widened the heating area and boosted the heat transfer efficiency, was selected based on the size of the drying device. Based on the thermal properties of air in the standard state, the following formula can be used to determine the power of the heating tube:

(1) $$P=\frac{\left(T_{2}-T_{1}\right)\;\cdot \;\left(C_{p}\times \rho \times V\right)\;\cdot \;t}{3600},$$

where *P* is the heating tube power, kW; *T*_1_ is the airflow temperature before heating, °C; *T*_2_ is the airflow temperature after heating, °C; *C_p_* is the specific heat of air, 1.003 kJ/(kg·K); *ρ* is the density of air, 1.293 kg/m^3^; *V* is the internal volume of the drying oven, m^3^; and *t* is the working time of the heating tube, h.

The necessary heating tube power was determined to be approximately 0.14 kW. The single-ended finned heating tube with a pipe diameter of 12 mm, outer diameter of 24 mm, and power of 0.40 kW was selected after determining the heat loss, heat insulation treatment, and size of the heating tube.

#### 2.3.3. Supply Fan Selection

The choice of fan has a significant impact on how well the materials are dried because it controls the airflow and performance of the drying room [22]. Two axial flow fans were chosen for the combined infrared and hot air-drying system based on temperature and humidity control. The amount of air that the drying device needed to function determined the output power of hot air circulation. The Q airflow rate of the drying chamber can be calculated using the following formula:

(2) $$Q=v\times \pi r^{2},$$

where *Q* is the airflow, m^3^/s; *r* is the fan radius, m; and *v* is the airflow velocity, m/s.

The calculated volume flow rate was 0.064 m^3^/s; that is, the required motor power, *P_f_*, was calculated as follows:

(3) $$P_{f}=Q\times P_{0}\times \eta \times 3600,$$

where *P_f_* is the fan power (W); *P*_0_ is the pressure difference between the fan outlet and inlet; and η is the fan efficiency.

Based on temperature and humidity control, it was determined that the fan power of the combined infrared and hot air-drying equipment was 6.3 W. It was essential to select a fan that was corrosion- and high-temperature-resistant owing to the dryer’s high temperature and humidity. A magnesium alloy frame, all-metal-blade, flame-retardant, and high-temperature-resistant axial flow fan (1238HA2, Shanghai Quanrui Electrical Equipment Co., Ltd., Shanghai, China) with a power of 16 W and speed of 2700 RPM was selected, considering safety performance, wind transportation, and installation size. The fan had a radius of 60 mm.

#### 2.3.4. Rack and Tray Design

The material rack, which was attached to the bottom of the drying box, was composed of 304 stainless steel walls. The dimensions of the material rack were 400 mm × 320 mm, including the size of the heating plate. The height of the rack was fixed at 500 mm. There was a 30 mm gap between the carbon fiber infrared radiant electric heating plate and the tray. The top-to-bottom design of the five-layer material tray may have increased the amount of drying processing and broadened the sea buckthorn processing scale.

A material tray of the high-and-low variety was chosen. The screen was mounted on the tray, and the sea buckthorn was placed on it to dry. Near the edge of the screen, between the screen and tray, was a group of magnetic suction gaskets. When the tray was placed down, it tilted to the side where the screen was raised, causing juice from the sea buckthorn to flow to the lower side because of the height difference. This effectively separated the sea buckthorn from the water and ensured sufficient quality of the dried sea buckthorn.

#### 2.3.5. Humidification and Dehumidification Device Selection

To increase the relative humidity in the drying chamber, existing drying equipment has frequently used wet curtain humidification. However, this method has drawbacks, including a long delay and slow response time [12]. To avoid the low temperature and high humidity of wet curtain humidification, the combined infrared and hot-air drying equipment based on temperature and humidity control used steam humidification. To avoid water vapor condensation, hot steam was supplied to the drying area. Avoiding the situation in which the external humidity is unstable makes it impossible to correctly control the humidity in the drying chamber. The KA1238HA2-HTR130 AC220 V all-metal cooling fan, high-temperature-resistant axial flow fan, and energy-saving stainless steel steam generator (Xi’an Jiasman Technology Development Co., Ltd., Xi’an, China) used in the combined infrared and hot-air drying device with temperature and humidity control had a combined thermal efficiency of 98%, a rated working pressure of 0.4 MPa, a rated evaporation capacity of 4 kg, a saturated steam temperature of 151 °C, and an adjustable medium humidity range of 0% to 95%. The relative humidity of the air in the drying room was precisely managed by an axial fan and steam generator working in tandem.

### 2.4. Control System Design

The machine interaction, data storage, control and decision, and communication modules were all parts of the combined infrared and hot-air drying device’s control system. This was based on temperature and humidity regulation. During the drying process, the control carbon fiber infrared radiation plate, exhaust fan, internal circulation fan, and electric heating tube were monitored, and the air temperature and humidity in the drying chamber were adjusted. To implement the multi-stage temperature and humidity control drying process, the exhaust mechanism and temperature control unit controlled the mechanical construction. The infrared board temperature monitoring and control system, medium temperature monitoring and control system, medium relative humidity monitoring and control system, material temperature monitoring system, circulating fan, and exhaust fan control system were the five functional systems that comprised the machine control system. Each system used the Modbus protocol to connect to the host. Finally, the monitoring and control function was completed.

#### 2.4.1. Human–Computer Interaction Platform Interface Design

The touch screen MT6071iE (Wilentong Technology Co., Ltd., Shenzhen, China), which is the host of the control system of the human–computer interaction platform, was selected for the study. The platform was used to complete all the drying device parameter input, reading, detection, control, and other operations. The touch screen had an 800 × 480 resolution. Three COM ports were used to connect the touch screen to each system (2 RS-485 and 1 RS-232), enabling the development of the human–machine interaction interface. The temperature controller and control module were connected via the Modbus RTU protocol via an RS-485 serial connector. Through AD conversion, software filtering, and conversion into decimal data, the temperature detected by the temperature sensor was transformed into electrical signals. The control module fed information back to the touch screen so it could read the temperatures of the drying chamber, material, and electric heating plate for carbon fiber infrared radiation. The temperatures of the finned single-head electric heating tube and carbon fiber infrared radiant electric heating plate were simultaneously regulated by the PID control program in accordance with the temperature controller’s preset value. To keep track of the regular operation of the axial and exhaust fans, one RS-485 serial port interacted with the PWM driver module, PWM acquisition module, and frequency converter. To read the temperature and humidity in the drying chamber and control the exhaust fan, an RS-232 serial port was connected to the temperature and humidity sensors.

#### 2.4.2. Medium Temperature Monitoring and Control

A PT100 temperature sensor (Shenzhen Haodu Technology Co., Ltd., Shenzhen, China) was used to measure the temperature in the drying chamber during the drying process, and the sensor accuracy was 0.1 °C. This was because the drying temperature for sea buckthorn is typically 50–80 °C [23], thus preventing the quality deterioration that results from drying at high temperatures. The temperature sensor in the drying oven was located in the upper air intake to reduce the impacts of wind speed and the heating equipment. To determine the current temperature in the drying oven, the temperature sensor was linked to the sensor input pin of the temperature controller. Data were transferred using the Modbus protocol to the human–computer interface for full storage and real-time display. The temperature setting value was transmitted via the human–computer interface to the temperature controller using the Modbus protocol, which then activated the relay to regulate the electric heating tube and carbon fiber infrared heating film.

#### 2.4.3. Medium Humidity Monitoring and Control

The humidity sensor of the drying chamber medium was an OMEGA HX71 humidity sensor (Shanghai Sibaiji Instrument System Co., Ltd., Shanghai, China), with an accuracy of 0.35% RH. The humidity and temperature sensors were positioned in the same horizontal position in the upper air inlet of the drying unit to ensure measurement accuracy. The humidity sensor sent relative humidity information via the Modbus RTU to the human–computer interface for storage and display. Based on the difference between the specified and actual humidity values, the humidity sensor assessed whether the exhaust fan was properly operating. The axial fan received the command via the Modbus protocol. The humidity sensor measured the relative humidity in the drying chamber during the drying of the sea buckthorn. The high moisture content of the sea buckthorn caused the surface moisture to evaporate into the drying chamber during the early drying phase, which in turn caused the humidity in the drying chamber to continuously rise. When the relative humidity exceeded a predetermined level, the exhaust fan was activated, which pushed the humidity outside. Then, the exhaust fan was turned off, and the humidifier was turned on when the humidity sensor reading fell below the predetermined level.

#### 2.4.4. Material Temperature Monitoring and Control

The temperature of the material was monitored using a PT100 temperature sensor (Shenzhen Haodu Technology Co., Ltd., Shenzhen, China). The sensor had a 1.47 mm diameter and a 0.10 °C precision. To prevent the sensor’s influence on the material’s heat transmission, the needle probe’s 10.54 mm length was encased in a heat shrink tube. The resistance value of the temperature sensor measured the change in temperature of the material, which was then translated into a change in the resistance value. This was then translated into a change in the voltage signal. The output end of the temperature transmitter was connected to the I/O pin of the PIC16F1947 single-chip microcomputer module, and an A/D converter transformed the analog signal into a digital signal. The Modbus ASCII protocol was used by the single-chip computer to provide temperature data to the human–computer interface for storage and display.

## 3. Analysis and Optimization of Flow Field in Air Distribution Chamber

After numerical modeling of the dryer, the accuracy of the model was assessed by comparing it with the prototype. The primary and secondary spoilers were fitted in the airflow distribution chamber using a CFD simulation to achieve uniform air input in the drying chamber.

### 3.1. Numerical Modeling and Meshing

**Figure 2:**
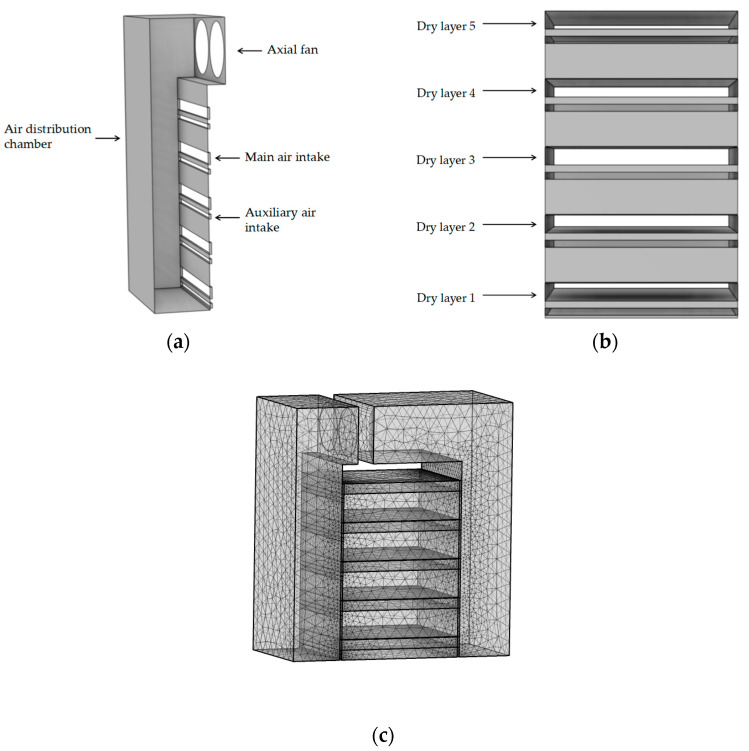
(**a**) Numerical model of airflow distribution chamber; (**b**) Number of drying chamber layers; (**c**) Dryer mesh division diagram.

Figure 2a shows the numerical model of the airflow distribution chamber of the dryer. The main air inlet measured 27 cm × 2.5 cm. The secondary air inlet was 27 cm × 1 cm in size, and the airflow that entered it dried the bottom of the material through the holes in the material tray. Given that it had little impact on the flow field in the drying chamber, the secondary air inlet was not considered in this study. According to Figure 2b, the drying chamber was divided into five tiers, numbered 1, 2, 3, 4, and 5. In this study, numerical simulations were performed using COMSOL Multiphysics 6.0 software. The program included all aspects of the simulation, such as design geometries, assessment, and analyzing the results. To achieve convergence, a coarser mesh was chosen, with a total mesh of approximately 385278, as illustrated in Figure 2c.

### 3.2. Control Equations and Simplifying Assumptions

The N-S equation describes the conservation of momentum of a viscous incompressible fluid. It is typically used to simulate the flow of Newtonian fluids. There is a balance between the rate of change of momentum acting on the fluid element online and the sum of external forces, reflecting the fundamental laws of viscous fluid flow [24]:

(4) $$\frac{\partial }{\partial_{t}}\left({\rho u}_{i}\right)+\frac{\partial }{\partial x_{j}}\left({\rho u}_{i}u_{j}\right)=\frac{\partial }{\partial x_{j}}\left[-{p\delta }_{\mathrm{ij}}+\mu \left(\frac{\partial u_{i}}{\partial x_{j}}+\frac{\partial u_{j}}{\partial x_{i}}\right)\right]+{\rho g}_{i},$$

where ρ is the fluid density; $x_{j}$ is the coordinate component in the flow field along direction j (m); $u_{j}$ is the average relative velocity component in the flow field along direction j (m/s); δ is the Kronecker increment; μ is the dynamic viscosity (kg/ms); and g is the gravitational acceleration (m/s^2^).

The most used turbulence computation approach is the RANS equation, and this time, the conventional k-turbulence model was selected to solve the equation, as in [25].

(5) $$\rho \left(u\cdot \nabla \right)k=\nabla \cdot \left[\left(\mu +\frac{\mu_{T}}{\sigma_{k}}\right)\nabla_{k}\right]+P_{k}-\rho \varepsilon ,$$

(6) $$\rho \left(u\cdot \nabla \right)\varepsilon =\nabla \cdot \left[\left(\mu +\frac{\mu_{T}}{\sigma_{\varepsilon }}\right)\nabla_{\epsilon }\right]+C_{\varepsilon 1}\frac{\varepsilon }{k}P_{k}-C_{\varepsilon 2}\rho \frac{\varepsilon^{2}}{k},$$

(7) $$\mu_{T}={\rho C}_{\mu }\frac{k^{2}}{\varepsilon },$$

(8) $$P_{k}=\mu_{T}\left[\nabla_{u}:\left(\nabla_{u}+{\left(\nabla_{u}\right)}^{T}\right)\right],$$

where k is the turbulent kinetic energy (m^2^/s^2^); μ is the viscosity (Pa∙s); $\mu_{T}$ is the turbulent kinematic viscosity (m^2^/s); $C_{\varepsilon 1}$ and $C_{\varepsilon 2}$ are the turbulence model coefficients;${\;\sigma }_{\varepsilon }$ and $\sigma_{k}$ are the kinetic energy and Prandtl number of kinetic energy dissipation, respectively; ε is the turbulent kinetic energy dissipation rate (m^2^/s^3^); and $C_{\mu }$ is the empirical constant coefficient.

The following assumptions were made in this experiment to eliminate unnecessary computational processes and maximize efficiency while maintaining accuracy:

(1) The drying medium air was a fully developed flow and regarded as an incompressible gas.

(2) The influence of the material tray on the airflow was excluded from the analysis.

(3) The inner and outer walls of the dryer were adiabatic, and no heat exchange occurred with the outside environment.

### 3.3. Boundary Conditions and Evaluation Indexes

The inlet boundary of the system was established under the presumption that the airflow direction was parallel to the boundary. The design of the axial fan indicated that a single fan may provide 150 m^3^/h of airflow or 0.0417 m^3^/s flow rate, with a medium (5%) level of turbulence intensity and a geometry-based turbulence length. Thereafter, the backflow was inhibited, and the outlet boundary condition was adjusted to pressure. The typical wall function approach was used to adjust the wall boundary condition, which was set to zero slip. The velocity deviation ratio, E, and velocity inhomogeneity coefficient, M, were used to assess the distribution of flow velocity in each drying layer of the drying chamber and the uniformity of the distribution of flow velocity, respectively, as follows [26]:

(9) $$E=\frac{\left|\bar{V_{L}}-\bar{V_{a}}\right|}{\bar{V_{a}}}\times 100\%.$$

### 3.4. Model Validation

**Figure 3:**
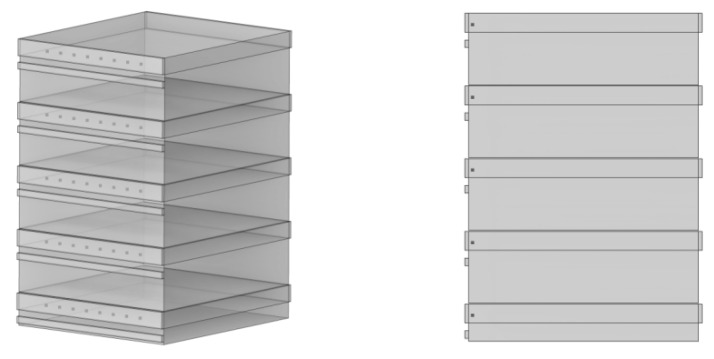
Dryer inlet air speed measurement points.

The accuracy of the built-in numerical model of the dryer needed to be confirmed to generate accurate simulation results. The actual values were measured using a hot-wire anemometer (TES-1304, TES Electronics Industry Co., Ltd. Taiwan, China). Meanwhile, the simulated values were measured using the point-probe tool of COMSOL Multiphysics software. A temperature of 75 °C was used for the drying tests. Prior to measurement, the machine was turned on for 10 min to preheat, and the wind speed measurement was initiated once the wind speed had stabilized. As shown in Figure 3, according to the actual placement position of the material, the first layer was taken as an example. The X, Y, and Z coordinates of the first point are taken as 0.047, 0.13, and 0.034 m, respectively, according to the actual placement position of the material, thereby ensuring uniformity. The wind speed measurement point was set, and eight locations were measured in a straight line with an X-axis separation of 0.025 m between the neighboring points of the same drying layer. The Z coordinate values of each drying layer measurement point were 0.034, 0.129, 0.224, 0.319, and 0.414 m, respectively, owing to the drying layer’s 0.095 m center distance.

**Figure 4:**
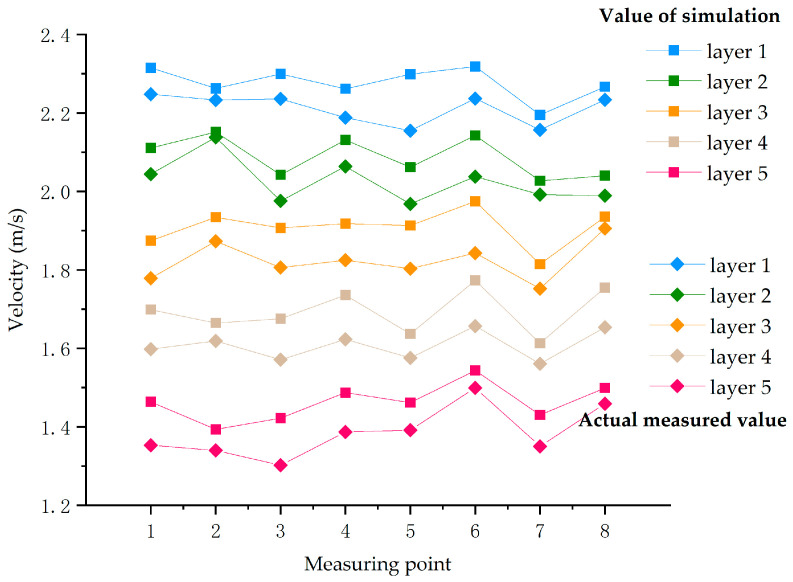
Comparison of real and simulated wind speed measurement results.

When the measurements were conducted, the drying box door was opened, and the anemometer was positioned at the predetermined location. The door was then closed to ensure sealing. When the wind speed displayed on the LCD screen was stable, data collection commenced. The anemometer’s averaging function was used to read the average value of the wind speed, and to ensure the accuracy of the measurement results, the wind speed sensor needed be positioned in line with the air outlet. Figure 4 shows a comparison of the actual and simulated wind speed measurement data. The simulated value was typically higher than the measured value because the simulation excluded the wind resistance of the items in the drying oven and the sealing state, which caused some loss of wind energy. The trends in the actual and calculated velocities were similar, and the wind speed steadily decreased from Drying Layer 1. The numerical model could be used later to offer a direction for flow field optimization because the highest difference between the actual and simulated wind speeds in the same drying layer was 7.10%, which satisfied the confidence criteria (10%).

### 3.5. Flow Field Analysis and Optimization

The velocity of each drying layer was found to decrease layer after layer during the preliminary model validation. This did not meet the requirement for drying uniformity. Consequently, the velocity flow field of the airflow distribution chamber needed to be optimized to achieve uniform drying.

**Figure 5:**
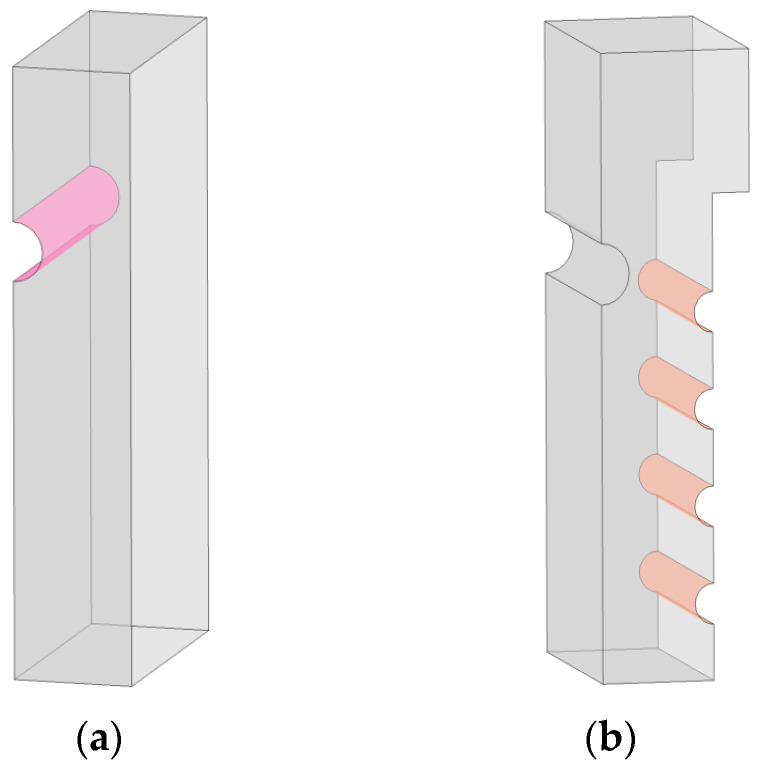
Half-cylinder spoiler on left side (**a**) and half-cylinder spoiler on right side (**b**).

**Figure 6:**
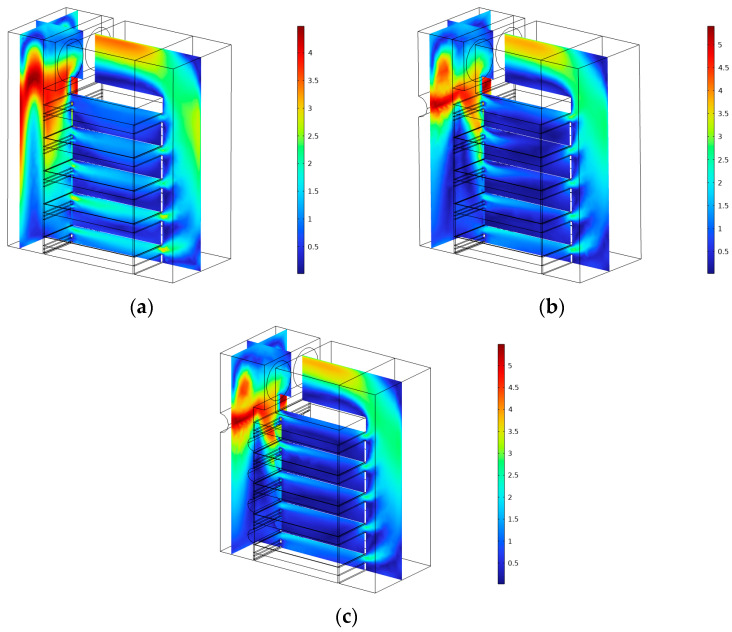
Velocity simulation rainbow graph before and after optimization. (**a**) Velocity simulation section diagram before spoiler installation. (**b**) Velocity simulation section diagram after semi-cylinder spoiler installation on the left. (**c**) Velocity simulation section diagram after semi-cylinder spoiler installation on the right.

Dai et al. [27] found that the addition of a semi-cylindrical spoiler on the left side of the air distribution chamber had a positive effect on the uniformity of the inlet air velocity of each drying layer. To explore the most appropriate optimization method, this study first placed a half-cylindrical spoiler with radius r = 3 across the entire air distribution chamber on the left side of the air distribution chamber, at the center of circle z = 40, as shown in Figure 5a. The remaining parameters remained unchanged. The boundary probe function of COMSOL Multiphysics software was used to obtain the average velocity of each drying layer inlet. The velocity simulation before and after installation of the spoiler is shown in Figure 6a,b.

The simulation comparison diagram showed that, prior to installation of the semi-cylindrical spoiler, a portion of the drying gas entered the airflow distribution chamber, collided with the inner wall, then flowed downward and converged into Drying Layer 1 at the bottom. As a result, the airflow velocity of this drying layer was excessive, whereas the uppermost drying layer received little airflow. The maximum velocity deviation ratio of each drying layer decreased from 26.68% to 13.17% after installing the spoiler, as shown by the probe results. This was because the semi-cylindrical spoiler structure forced some of the airflow into the uppermost drying layer and interfered with the vertical downward process of the airflow, thereby avoiding the convergence phenomenon at the bottom.

Four semi-cylindrical spoilers with a radius of 2 cm were installed at z = 7.5, 17, 26.5, and 36 cm above the air inlet of Drying Layer 14 on the right side of the air distribution chamber, as shown in Figure 5b. The velocity simulation results after installation are shown in Figure 6c. This was performed to further optimize the flow field. The half-cylinder structure scrambled and redistributed the airflow gathered at the air inlet of the drying layer after the addition of the right-side spoiler, allowing the airflow to enter each drying layer more uniformly. The right side of the airflow distribution chamber could only be installed in a limited number of positions owing to the air inlet. Therefore, the next step was to maintain the right spoiler’s size and position while using numerical simulation to change the left half-cylinder spoiler’s position and size to determine the most suitable design. The research procedure is presented in Table 1.

The maximum speed deviation ratio at various radii exhibited a trend of first decreasing and then increasing as the circle center coordinates increased. The minimum value of 0.88% was found at the circle center coordinates z = 43 and radius r = 3, making this the most appropriate optimization strategy.

## 4. Experimental Research and Analysis

### 4.1. Test Materials

Fresh sea buckthorn fruits were picked from 170 Regiment, 9th Division, Xinjiang Production and Construction Corps, in January 2022. Fruit without mechanical damage and of uniform size (average weight 0.52 ± 0.10 g, long axis length 15.00 ± 0.50 mm, short axis length 9.00 ± 0.50 mm) were chosen. A moisture content of 81.91 ± 0.50% was present.

### 4.2. Test Method

The drying temperature used in the preliminary experiment was 75 °C, and 10% relative humidity was used to compare the two humidification schemes with and without humidification. Approximately 100 g of sea buckthorn fruit was spread out on the tray for the drying process and removed approximately every 30 min. The water content was dried to less than 17% before the weight was determined using a balance (JA21002, Shanghai Shunyu Hengping Scientific Instrument Co., Ltd., Shanghai, China, precision 0.01 g). Following this process, the sample was removed, brought to room temperature, and sealed in a bag.

#### 4.2.1. Drying

The drying of sea buckthorn is described according to the water ratio–drying time and drying rate–dry base moisture content curves [28].

(10) $$MR=\frac{M_{t}}{M_{0}},$$

(11) $$DR=\frac{M_{t_{1}}-M_{t_{2}}}{t_{2}-t_{1}},$$

where *MR* is the water ratio; *M_t_* is the dry base moisture content at time t, g/g; *M*_0_ is the initial dry base moisture content, g/g; *t*_1_ and *t*_2_ are drying times, min; *M_t_*_1_ and *M_t_*_2_ are the moisture contents of the dry base at drying times t_1_ and *t*_2_, respectively, g/g; and *DR* is the drying rate of the material between *t*_1_ and *t*_2_ during the drying process, g/(g·h).

The Weibull distribution function was used to estimate the effective diffusion coefficient, *D_eff_*, in the drying process of materials [29].

(12) $$MR=\exp\left[-{\left(\frac{t}{\alpha }\right)}^{\beta }\right],$$

where *MR* is the water ratio; *M_t_* is the dry base moisture content at time t, g/g; *M*_0_ is the initial dry base moisture content, g/g; *t*_1_ and *t*_2_ are drying times, min; *M_t_*_1_ and *M_t_*_2_ are the moisture contents of the dry base at drying time*s t*_1_ and *t*_2_, respectively, g/g; and *DR* is the drying rate of the material between *t*_1_ and *t*_2_ during the drying process, g/(g·h).

(13) $$D_{\mathrm{eff}}=\frac{D_{\mathrm{cal}}}{R_{g}}=\frac{r^{2}}{\alpha Rg},$$

where *D_cal_* is the water effective diffusion coefficient estimated during the sea buckthorn drying process, m^2^/s; *r* is the equivalent radius of sea buckthorn; and *R_g_* is a parameter related to the geometrical size of the material, which is 13.1 m^2^/s for the spherical sea buckthorn fruit.

#### 4.2.2. Specific Energy Consumption (SEC)

The energy consumption in the drying process of materials can be calculated according to the specific energy consumption (SEC) [30], as shown in Equation (14):

(14) $$SEC=\frac{1000W}{m_{0}\phi_{0}-m_{i}\phi_{i}},$$

where *SEC* represents the unit energy consumption of an experiment, kW·h/kg; *W* represents the difference in electrical representation before and after the experiment, kW·h; *m*_1_ and *m*_2_ represent the initial and final mass of the experimental material, respectively, g; and *φ*_1_, and *φ*_2_ represent the wet base moisture content of the material at the beginning and end of drying, respectively.

#### 4.2.3. Color Difference Degree

Color was quantitatively determined using a color difference meter (Beijing Mingyang Technology Development Co., Ltd., Beijing, China). L* (brightness), a* (red/green), and b* values (yellow/blue) were measured to calculate the total color difference (Δ*E*) of the materials [31].

(15) $$\Delta E=\sqrt{{\left(L^{*}-L_{0}{}^{*}\right)}^{2}+{\left(a^{*}-a_{0}{}^{*}\right)}^{2}+{\left(b^{*}-b_{0}{}^{*}\right)}^{2}}$$

#### 4.2.4. Browning Degree

The browning degree was determined according to Deng et al. [32]. Sea buckthorn powder (2.0 g) was evenly ground with 20 mL of distilled water and centrifuged at 4 °C at 10,000 r/min for 30 min. The absorbance of the clear liquid with a degree of browning was measured at 420 nm using a spectrophotometer (Beijing Purkinje General Instrument Co., Ltd., Beijing, China).

#### 4.2.5. Determination of Rehydration Ratio

According to the method of Wang [33], a glass containing deionized water was placed in a 40 °C water bath. When the temperature of the deionized water was constant, a certain mass of dried sea buckthorn was added to the deionized water and the timer was started. After 12 h, the sample was removed and drained, and the water on the surface of the material was wiped with absorbent paper. The rehydration ratio was calculated according to Equation (16):

(16) $$RR=\frac{m_{2}}{m_{1}}$$

#### 4.2.6. Ascorbic Acid Retention Rate

The titration method of Wang [34] for the determination of Vitamin C content was slightly modified in this study. Pulp (1.0 g) was weighed into a mortar and a small amount of 20 g/L oxalic acid solution was added. The resulting mixture was ground into pulp in an ice bath, transferred to a 100 mL volumetric bottle, rinsed with 20 g/L oxalic acid solution, and the rinse solution was poured into the volumetric bottle. Then, 20 g/L oxalic acid solution was added to fix the volume to scale, the mixture was shaken well, and then centrifuged at 8000 r/min for 10 min. To avoid the influence of the color of the sample solution, 2,6-dichlorophenol indophenol reverse titration was used to calculate the ascorbic acid content of the sample. The ascorbic acid content of the sample was expressed per dry base (mg/100 g DW) and calculated according to Equation (17) for direct comparison with the fresh sample. The ratio between the measured value and that of the fresh sample indicated the retention rate of ascorbic acid.

(17) $$A=\frac{c\cdot V_{1}\cdot V_{2}}{V_{3}\cdot W}\cdot 100$$

#### 4.2.7. Data Processing

IBM SPSS Statistics 25 was used to analyze the drying and quality analysis test data. S-N-K multiple testing was used for variance analysis (*p* < 0.05), and the charts were processed using Excel 2016.

### 4.3. Test Results and Analysis

#### 4.3.1. Drying Kinetics

**Figure 7:**
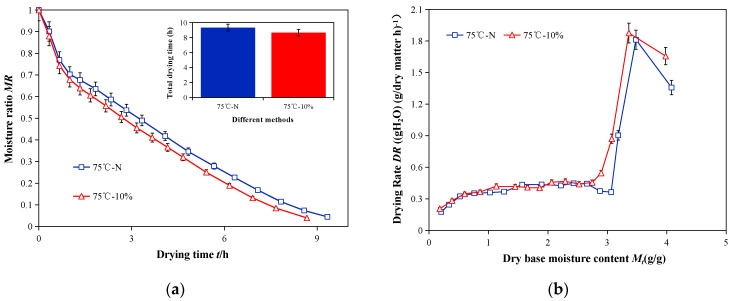
Kinetics curves of different drying processes. (**a**) Curve of drying water ratio of sea buckthorn with drying time under different drying methods. (**b**) Curves of drying rate of sea buckthorn with dry base moisture content under different drying methods.

The effects of humidification and non-humidification on the drying kinetics of sea buckthorn are shown in Figure 7. The drying kinetics were significantly altered by humidification, which shortened the drying time by 7.18% (Figure 7a). This effect may have been because raising the relative humidity can hasten the material’s temperature rise and the inward and outward movement of water molecules in the sea buckthorn [35]; thus, the higher relative humidity prevented the surface crust phenomenon on sea buckthorn. This made it simpler for water to diffuse from the inside to the outside and accelerated drying [36]. The drying rate first increased and then decreased, as shown in Figure 7b. In the early stage, the sea buckthorn transitioned from a frozen state to a hot processing state, which had a lower internal temperature and slower drying rate, and this may have been the cause of the rising rate. In the later stage, the sea buckthorn fruit heated up quickly, and the drying rate accelerated. In the later stage, the drying rate decreased with a decline in the dry base moisture content. Furthermore, the water spread from the inside to the outside during the drying stage at a constant rate. The image shows that raising the relative humidity could speed up the drying process, which may be because sea buckthorn grows more readily when there is high humidity. Sea buckthorn dried out more quickly in the beginning because the heat was focused on draining the surface water of the plant [37]. When comparing the drying of sea buckthorn with and without humidification, the drying rate of the sea buckthorn without humidification was lower than that with humidification. This may have been the result of water evaporating more quickly in a low-humidity environment, which caused the sea buckthorn fruit’s skin to shrink and form a crust on its surface, preventing water from dripping through and extending the drying process. The difference between the cut-off moisture concentration and surface moisture concentration of the material decreased as the drying process progressed. This decreased the heat and mass transfer power, as well as the drying rate [38]. The effective water diffusion coefficients of the sea buckthorn under the two conditions of combined infrared and hot-air drying with and without humidification, respectively, were calculated using the Weibull distribution function. They were found to be 1.23 × 10^−8^ and 1.12 × 10^−8^ m^2^/s, which also demonstrated that a high-humidity environment could provide the sea buckthorn with a higher heat source to facilitate the movement of water molecules. This, in turn, promoted the diffusion of water molecules.

#### 4.3.2. Drying Quality

Color brightness has been shown to have a significant influence on consumers’ desire to purchase agricultural products, and color is a key determinant of dry quality assessment [39]. The color contrast between the sea buckthorn dried with and without humidification is shown in Table 1. Table 1 shows that the color difference, Δ*E*, diminished after humidification, and the humid environment improved the color of the sea buckthorn after drying. This was consistent with the research of Ju et al. As a result of the humidification treatment, the brightness value, L*, and blue/yellow value, b*, of the dried sea buckthorn were higher than those of fresh samples, which may have been related to the reduction in drying time, which prevented oxidation [6]. The early high humidity level may have caused the material to heat up more quickly, inhibiting polyphenol oxidase and peroxidase activities and preventing the development of enzymatic browning [40]. The browning index also showed that sea buckthorn dried with infrared and hot-air treatment under humidification had a reduced degree of browning, which meant that the humidification treatment could prevent the development of enzymatic browning.

The vitamin C level in sea buckthorn is high [5], but it decreases through drying. Therefore, it is important to prevent vitamin C from degrading during the drying process. The results in Table 2 demonstrate that the vitamin C retention rate of dried sea buckthorn increased by 6% after humidification compared to that without humidification. This increase in the vitamin C retention rate may have been attributable to the reduced drying time after humidification. In addition to Geng et al.’s discovery that vitamin C retention is positively linked to drying time [40], heat loss of vitamin C was prevented.

**Figure 8:**
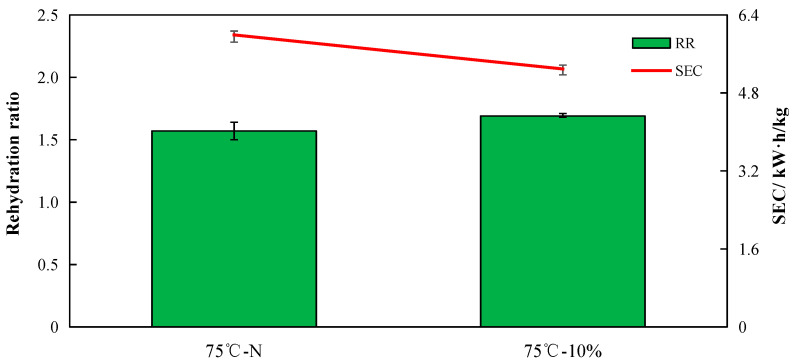
Specific energy consumption (SEC) and rehydration ratio (RR) of different drying processes.

The rehydration of agricultural products that have been dried may indicate how heat treatment affects the structure of the materials after drying [41]. Agricultural items that have been dried under optimum conditions can be rehydrated to their original fresh state. However, given that drying is an irreversible process, the rate of rehydration can indicate the extent to which different processing techniques have harmed the structure [42]. The rehydration ratio of sea buckthorn dried with and without humidification is shown in Figure 8. Our findings demonstrated that the rehydration ratio following humidification was higher than that in the control group, supporting the hypothesis that early humidification opened the pores and encouraged the flow of water vapor. Sea buckthorn pores could be opened via humidification, which resulted in a higher rehydration ratio. Zhang et al. [36] discovered a similar effect when humidifying and drying berry materials, and the rehydration ratio of berries under humidification treatment was significantly higher than that without humidification.

Energy consumption is a significant factor to consider when weighing the advantages and disadvantages of drying because drying is an energy-consuming process. For the industry to use less energy, energy consumption during drying needs to be substantially reduced [43]. The study findings demonstrated that humidification treatment minimized drying time and energy use (Figure 8). Compared with non-humidification treatment, humidification treatment was shown to use 11.02% less energy to remove one unit of water.

A combined infrared and hot-air drying technique based on temperature and humidity control and high-efficiency radiation heat transfer was provided as a solution to long cycle times and poor quality in sea buckthorn drying. By presenting this hot-air drying model as a potential high-efficiency and high-quality preservation technology for sea buckthorn drying, we hope to advance the development of research in the sea buckthorn drying sector. However, further research on the relative humidity management systems used in real-world production is required to understand how relative humidity affects drying kinetics and energy consumption.

## 5. Conclusions

This study developed a combined infrared and hot-air dryer based on temperature and humidity control to address the issues of lengthy drying process and low-quality dried product for sea buckthorn. Semi-cylindrical spoilers were introduced and paired with a computational fluid dynamics simulation to address the inconsistent speed of the inlet of each drying layer. The ideal velocity deviation rate was found to be 0.88% when the spoiler’s center coordinates were z = 43 and r = 3, according to an analysis of the simulation results for different sizes and positions of the spoiler. The drying time was shortened by 7.18% after drying with humidification during the experimental verification of sea buckthorn’s drying characteristics, and the plant’s actual water diffusion coefficient increased from 1.12 to 1.23 m^2^/s. The dried sea buckthorn had more vibrant color, it retained more vitamin C, and it had an improved rehydration ratio. In the future, we will investigate an adaptive control for drying with low temperature and high humidity in the initial stage and high temperature and low humidity in the final stage. In addition, an adaptive control strategy for drying sea buckthorn based on energy consumption, drying efficiency, fan quality, and the heating plate unit will be developed. Moreover, the software and hardware of an intelligent control system will be realized for precise judgment of the drying endpoint.

## Figures and Tables

**Table 1:** Study of the position and dimensions of the left half-cylinder spoiler.

Prioritization Scheme	Maximum Velocity (m/s)	Minimum Velocity (m/s)	Maximum Velocity Deviation Ratio
z = 38, r = 1	1.7124	1.3025	18.33%
z = 38, r = 2	1.7848	1.4591	14.13%
z = 38, r = 3	1.7737	1.3843	13.38%
z = 38, r = 4	2.0215	1.3417	27.43%
z = 39, r = 1	1.7126	1.2967	19.01%
z = 39, r = 2	1.7685	1.5148	11.02%
z = 39, r = 3	1.8009	1.4477	13.32%
z = 39, r = 4	2.0164	1.4304	24.54%
z = 40, r = 1	1.7045	1.3177	18.05%
z = 40, r = 2	1.5471	1.4226	5.02%
z = 40, r = 3	1.5905	1.3468	8.41%
z = 40, r = 4	1.7702	1.3231	18.28%
z = 41, r = 1	1.6966	1.2992	19.41%
z = 41, r = 2	1.7305	1.524	6.81%
z = 41, r = 3	1.7551	1.5864	7.24%
z = 41, r = 4	1.9825	1.5842	17.77%
z = 42, r = 1	1.7043	1.2841	20.21%
z = 42, r = 2	1.707	1.4702	9.30%
z = 42, r = 3	1.7205	1.6063	4.45%
z = 42, r = 4	1.8499	1.599	10.73%
z = 43, r = 1	1.7236	1.2799	20.55%
z = 43, r = 2	1.6845	1.461	9.87%
z = 43, r = 3	1.6539	1.6294	0.88%
z = 43, r = 4	1.6815	1.6125	5.39%
z = 44, r = 1	1.7334	1.3054	19.18%
z = 44, r = 2	1.7057	1.4264	12.17%
z = 44, r = 3	1.6856	1.543	5.90%
z = 44, r = 4	1.6939	1.5521	5.87%

**Table 2:** Drying quality of sea buckthorn under different drying methods.

Parameter	Fresh	Drying Methods	
		75 °C-N	75 °C-10%
	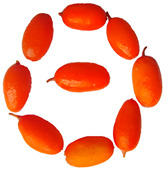	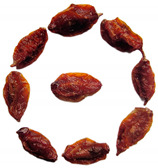	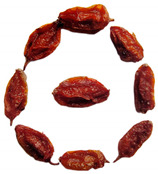
L*	57.14 ± 0.11 ^a^	38.47 ± 0.26 ^c^	39.28 ± 0.04 ^b^
a*	33.34 ± 0.22 ^a^	23.14 ± 0.03 ^b^	22.89 ± 0.06 ^c^
b*	54.83 ± 0.23 ^a^	35.59 ± 0.21 ^c^	39.76 ± 0.04 ^b^
Δ*E*	/	28.69 ± 0.31	25.60 ± 0.05
Browning index	0.09 ± 0.00 ^c^	0.34 ± 0.01 ^a^	0.26 ± 0.02 ^b^
(Abs/g d.m.)			
Vc retention rate	1.00 + 0.00 ^a^	0.24 + 0.07 ^c^	0.32 + 0.01 ^b^

Notes: different letters in the table reveal significant differences (*p* < 0.05) according to Duncan’s test.

## Data Availability

The data used to support the findings of this study can be made available by the corresponding author upon request.
